# Real-world Imatinib Mesylate Treatment in Patients with Chronic Myeloid Leukemia: The Importance of Molecular Monitoring and the Early Molecular Response

**DOI:** 10.1007/s00277-023-05189-3

**Published:** 2023-04-13

**Authors:** Amanda Pifano Soares Ferreira, Fernanda Salles Seguro, Andre Ramires Neder Abdo, Fernanda Maria Santos, Felipe Vieira Rodrigues Maciel, Luciana Nardinelli, Ricardo Rodrigues Giorgi, Antonio Roberto Lancha Ruiz, Milton Pifano Soares Ferreira, Eduardo Magalhaes Rego, Vanderson Rocha, Israel Bendit

**Affiliations:** 1 Hematology Clinic Oncoclinicas, Sao Paulo, Brazil; 2 Department of Hematology, Transfusion and Cell Therapy, University of Sao Paulo Medical School (HCFMUSP), Sao Paulo, Brazil; 3 Department of Hematology, Cancer Institute of Sao Paulo, University of Sao Paulo Medical School (ICESP), Sao Paulo, Brazil; 4 Laboratory of Medical Investigation in Pathogenesis and targeted therapy in Onco-Immuno-Hematology (LIM/31), Department of Hematology, Hospital das Clínicas HCFMUSP, Faculdade de Medicina, Universidade de Sao Paulo, Sao Paulo, Brazil; 5 Minas Gerais Federal University, Belo Horizonte, Brazil; 6 Hemato-Oncologia, DASA-Genômica, Sao Paulo, Brazil

**Keywords:** Chronic myeloid leukemia, *BCR::ABL1* transcript, early molecular response, profound molecular response, imatinib mesylate, real-world, Conflict of interest statement: There are no conflicts of interest to report.

## Abstract

**Introduction:**

Chronic myeloid leukemia (CML) is a clonal myeloproliferative disorder characterized by the Philadelphia (Ph) chromosome. After the introduction of imatinib mesylate (IM) in 2000, the natural history of the disease changed. Data on the treatment of CML with IM are from randomized clinical trials. Establishing whether these results can be reproduced or if caution is needed when extrapolating data to the general population with CML is essential.

**Objectives:**

To evaluate the molecular response (MR) in patients with chronic-phase CML (CML-CP) not included in clinical studies and correlate them with the responses obtained in clinical trials.

**Methods:**

Between January 2007 and January 2017, 227 patients newly diagnosed with CML-CP treated with IM as first-line treatment were included. This study is an observational, retrospective, and single-center study.

**Results:**

At a median follow-up time of 7.3 years, 60.3% of the 227 patients who started IM were still on IM. Early molecular response (EMR) at 3 and 6 months was achieved by 74.2% and 65%, respectively. The median time to a MMR was nine months. The MR4.0 and MR4.5 were 67.2% and 51.1%, respectively. The overall survival (OS), progression-free survival (PFS), and event-free survival (EFS) of the patients who exclusively used IM were 91%, 91%, and 85.1%, respectively.

**Conclusion:**

The results presented are similar to those described in prospective and randomized trials, demonstrating that the outcomes are reproducible in the real world. EMR at 3 and 6 months reflects better long-term responses, including higher rates of deeper molecular responses. Considering treatment costs, the absence of literature evidence of an impact on overall survival demonstrated by first-line second-generation tyrosine kinase inhibitors (TKIs), and the global OS of 85.8%, imatinib mesylate (IM) is still an excellent therapeutic option.

## Introduction

CML is a clonal myeloproliferative disorder of pluripotent hematopoietic stem cells, in which a reciprocal translocation occurs between chromosomes 9 and 22, t(9;22)(q34;q11.2), resulting in the Philadelphia (Ph) chromosome, which is responsible for the expression of an abnormal fusion protein with altered tyrosine kinase activity called *BCR::ABL1* [1]. The development and approval of TKIs for treating CML-CP have led to their becoming the first-line therapy for CML patients. The International Randomized Study of Interferon and STI571 (IRIS) is considered a reference clinical trial for CML-CP treatment since its fundamental modifications to the treatment resulted in advances in the prognosis and altered the disease's natural course [2]. Today, four TKIs have been approved for the first-line treatment of CML-CP: imatinib, dasatinib, nilotinib, and bosutinib.

The monitoring milestones of *BCR::ABL1* transcript levels at 3, 6, and 12 months determine whether the current treatment should be continued (optimal response), changed (failure/resistance), or carefully considered for continuation or change, depending on patients’ characteristics, comorbidities, and tolerance (warning). TKIs have improved patient outcomes to near-normal life and evolved into a chronic conditions with age-related comorbidities. There has been increasing focus on the quality of life, avoiding long-term organ toxicities, and identifying strategies to maximize the possibility of stopping TKI therapy (treatment-free remission - TFR) which is feasible for some patients with deeper response [3–9].

Data on TKI treatment for CML are from clinical trials in which frail or elderly patients with multiple comorbidities were generally excluded [10]. The main objective of this study was to compare the MRs obtained during the treatment of CML-CP with IM as the first line in patients not included in clinical studies with the responses seen in randomized clinical studies and to verify whether these results can be generalized or if caution is needed when extrapolating the data to the general population with CML. The patients were followed in a reference hematology service in a Brazilian public health hospital.

## Materials And Methods

### Patients and inclusion criteria

This is a single-center, retrospective study of patients diagnosed with CML-CP per World Health Organization (WHO) 2007 criteria between January 2007 and January 2017 at the Hospital das Clínicas at Faculdade de Medicina of the Universidade de Sao Paulo hematology service.

**Fig. 1:**
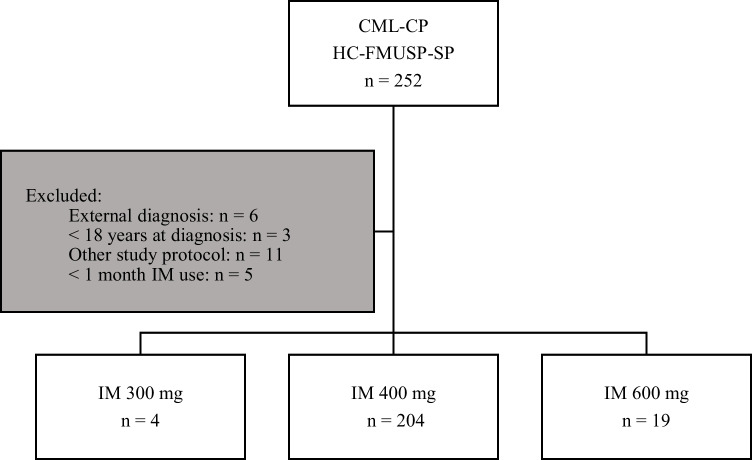
Study algorithm, patient enrollment, and treatment

The eligibility criteria were age greater than or equal to 18 years and use of IM as a first-line treatment regardless of the initial dose, exclusive diagnosis, and follow-up at our institution. Patients who did not use the medication regularly for at least one month or were inserted in research protocols were excluded (Figure 1).

The data was collected in a RedCap database. The study was approved by the ethics committee of the Hospital das Clínicas at Faculdade de Medicina of the Universidade de Sao Paulo.

### Molecular evaluation and therapeutic response

The *BCR::ABL1* transcript type was defined using the reverse transcription molecular response (RT-MR), and the molecular monitoring of *BCR::ABL1* transcript levels was performed using the quantitative reverse transcription molecular response (QRT-MR) technique as described elsewhere [11]. Patients underwent clinical and molecular follow-ups every three months in the first year of treatment or until they reached a MMR. For data collection, we considered *BCR::ABL1* results of samples collected at 3, 6, 12, and 18 months with a margin of one month for less or more from these milestones. Subsequently, patients were followed up every 3 to 6 months or according to clinical judgment. Molecular monitoring and therapeutic response were presented and analyzed according to the recommendation criteria of the ELN 2013 [12], but clinical decisions were by the follow-up period guidelines.

### Statistical analysis

The data were last updated in June 2019. Statistical analyses were performed using the R Core Team program (2020). The population characteristics are expressed descriptively. Pearson's χ-square and Fisher’s exact tests were used to compare molecular response rates. The differences in survival were estimated using the Kaplan–Meier method, and the differences between them and the cumulative rates of MRs were calculated using log-rank tests. The odds of MMR, MR4.0, and MR4.5 were calculated using the cumulative incidence process. PFS was defined as the time between the date of diagnosis and death from any cause or date of progression to the accelerated phase (AP) or blastic crisis (BC). EFS was defined as the time between the date of diagnosis and the date of death from any cause, the date of progression to AP or BC, the date of loss of MMR, the date of increase in the dose of IM, and the date of switching to a second line TKI, either due to loss of response, resistance or intolerance to IM, progression to AP or BC, and date of the last consultation. OS was defined as the time between diagnosis and death from any cause, date of the patient's last visit, or loss to follow-up. Statistical significance was defined when p <0.05.

## RESULTS

### Patient characteristics

Two hundred and twenty-seven patients were diagnosed with CML-CP. The median age was 49.6 years at diagnosis (range 18 to 89 years), with a slight male-to-female predominance of 1.1:1. The *BCR::ABL1* e13a2, e14a2, and e13a2/e14a2 transcripts were observed in 42% (n = 95), 52.8% (n = 120) and 5.2% of the patients (n = 12), respectively. The median time to start IM was 1.7 months (range 0 to 24.4 months). Five patients began treatment with INF-α because the diagnosis of CML-CP occurred during the gestational period. Still, after the pregnancy had concluded, IM was introduced at 400 mg daily. Ninety patients (39.7%) switched to second-line treatment for various reasons, the most common being primary resistance (no hematologic response or no complete cytogenetic response (CCyR) or no MMR, as categorized by ELN 2013) in 47.7% (43/90) and intolerance to treatment in 22.2% (22/90), in a median time of 1.2 years (range 0.2 to 10.4). A switch to dasatinib occurred in 86.7% (78/90), nilotinib in 11.1% (10/90), IFN-α in 1.1% (1/90), and hydroxyurea in 1.1% of patients (1/90). In 27 (11.9%) patients, the IM dose was increased to 600 mg, the leading cause of no cytogenetic response, in a median time of 1.1 years (range 0.3 to 10.4). The remaining characteristics of the patients are detailed in Table 1.

**Table 1:** Demographic, clinical, and laboratory characteristics of the 227 patients diagnosed with CML

**Sex**	** n = 227 (%)**
Male	118 (52)
Female	109 (48)
**Age – median**	49.6 (18-89)
***BCR::ABL1 *transcript**	
e13a2	95 (42)
e14a2	120 (52.8)
e13a2/e14a2	12 (5.2)
**Initial treatment**	
MI 300 mg	4 (1.8)
MI 400 mg	199 (87.6)
MI 600 mg	19 (8.4)
IFN-α	5 (2.2)
**Time start IM – median (months)**	1.7 (0-24.4)
**Second-line therapy switch**	
No	137 (60.3)
Yes	90 (39.7)
**Causes of therapy switching**	
Primary resistance	43 (47.7)
Intolerance to IM	20 (22.2)
Loss of any response to treatment	12 (13.3)
Progression disease	12 (13.3)
Inclusion in clinical trials	3 (3.4)
Not switched treatment	137 (60.3)
**Time to second-line therapy – median time (years)**	1.2 (0.2-10.4)
**Second line treatment**	
Dasatinib	78 (86.7)
Nilotinib	10 (11.1)
IFN-α	1 (1.1)
Hydroxyurea	1 (1.1)
**Increased IM dose to 600 mg**	
No	200 (88.1)
Yes	27 (11.9)
**Cause for increased IM dose**	
Loss of complete hematologic response	0 (0)
No cytogenetic response	22 (81.5)
No molecular response	5 (18.5)
**Time to increase IM dose – median time (years)**	1.1 (0.3-10.4)
***ABL1 *gene mutation**	
No	26 (11.5)
Yes	4 (1.8)
Not concluded	1 (0.4)
*ABL1* non-T315I gene mutation	3 (75)
**Suspension of therapy (protocol study)**	
No	211 (93)
Yes	16 (7)
**Third-line therapy switching**	
No	69 (76.7)
Yes	21 (23.3)
**Follow-up – median time (years)**	7.3 (0.4-12.4)

#### Early molecular response

We analyzed the incidence and the median time to reach the EMR at 3 and 6 months, as shown in Table 2. At 3 months, *BCR::ABL1* transcript levels ≤10% were achieved in 74.2% of patients (164/221) in a median time of 3.1 months, and at 6 months, 65% (134/206) reached transcript levels <1% (median: 6.2 months).

**Table 2:** Molecular responses by 3 and 6 months

PCR *BCR::ABL1* by 3 months					
n = 221	≤10%	74.2% (164)	>10%	25.8% (57)	Median time: 3.1 months
PCR *BCR::ABL1* by 6 months					
n = 206	<1%	65% (134)	≥1%	35% (72)	Median time: 6.2 months

**Fig. 2:**
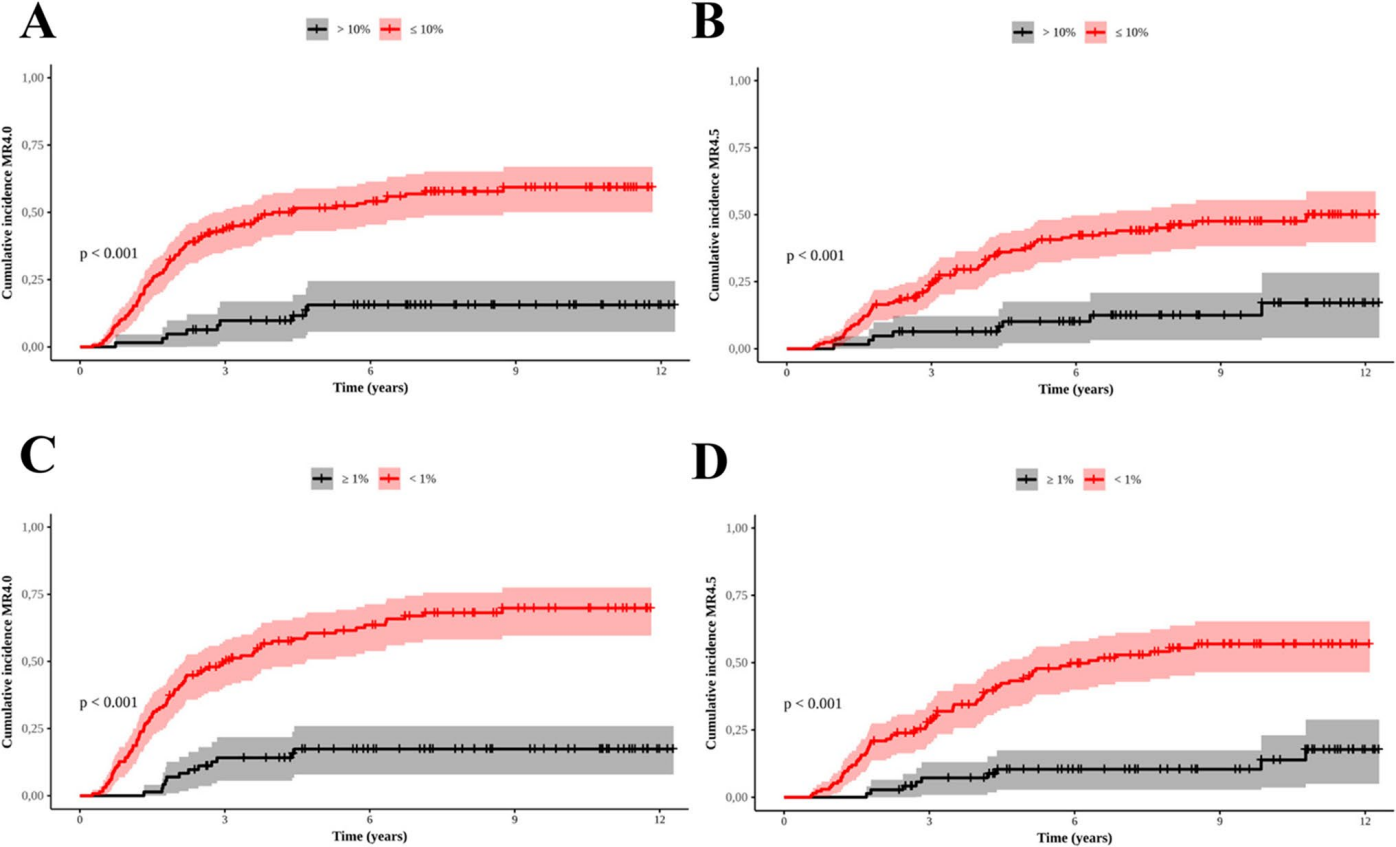
Cumulative incidence of MR4.0 and MR4.5 in patients treated with imatinib who reached EMR by 3 and 6 months. **A**: EMR by 3 months 10% and 10% and MR4.0; **B: **EMR by 3 months 10% and 10% and MR4.5; **C**: EMR by 6 months 1% and 1% and MR4.0; **D**: EMR by 6 months 1% and 1% and MR4.5. Red and black curves represent respectively those who did and did not reach EMR

**Fig. 3:**
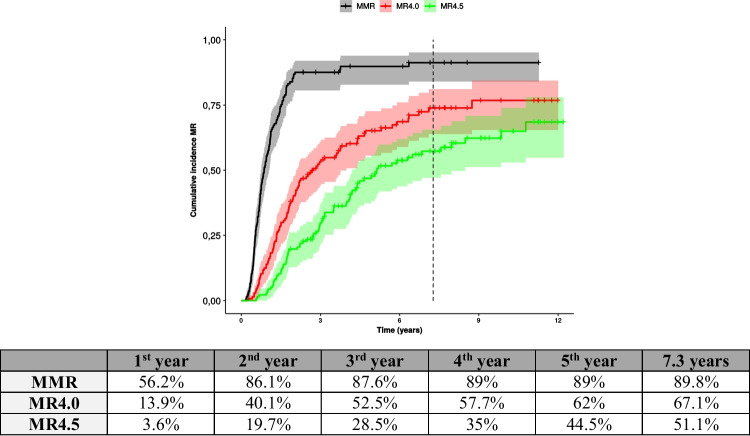
Cumulative incidence of major molecular response (MMR), and deep molecular response (MR4.0 and MR4.5)

Table 3 shows the results of the comparison between patients who achieved and did not achieve EMR at 3 and 6 months with MMR at 12 and 18 months, and deep molecular responses [MR4.0 (*BCR::ABL1* ≤0.01%) and MR4.5 (*BCR::ABL1* ≤0.0032%)]. Between those who reached EMR at 3 and 6 months and collected *BCR::ABL1* quantitative samples at 12 months, 69.5% (98/141) and 72.1% (93/129) reached MMR at these time points, respectively. Comparatively, for those who did not present EMR, 14.3% (6/42) and 17.3% (9/52) reached MMR at 12 months, respectively (p<0.001). In the group that got EMR at 3 and 6 months and collected *BCR::ABL1* quantitative samples at 18 months, 78.7% (100/127) and 81.5% (97/119) reached MMR at these benchmarks, respectively. While those who did not present EMR, 35.5% (11/31) and 36.8% (14/38) got MMR at 18 months, respectively (p<0.001). Among patients with an EMR at 3 months, 54.9% (90/164) achieved MR4.0 in a mean time of 6.3 years (SD: 0.4), and 14% (8/57) who did not reach EMR at 3 months was 10.6 years (SD: 0.44) (p<0.001) (Figure 2A). The mean time to MR4.5 for 42.7% (70/164) patients who reached EMR at 3 months was 8.0 years (SD: 0.38), and for 12.3% (7/57) patients who did not achieve the same molecular response at 3 months, was 11.0 years (SD: 0.4) (p<0.001) (Figure 2B). For 64.2% (86/134) patients who achieved an EMR at 6 months, the mean time to reach MR4.0 was 5.4 years (SD: 0.43) and 7.2 years (SD: 0.42) for 50.7% (68/134) who got MR4.5. For 16.7% (12/72) who did not achieve an EMR in that time, the mean time to MR4.0 was 10.4 years (SD: 0.44), and for 12.5% (9/72), the mean time to MR4.5 was 11.1 years (SD: 0.35), respectively (Figure 2C and D). Molecular responses increased progressively throughout the follow-up, and the percentage of patients who achieved deeper molecular responses was higher in the first two years of IM use (Figure 3).

**Table 3:** Evaluation and comparation of early molecular response by 3 and 6 months with major molecular response by 12 and 18 months*, and MR4.0 and MR4.5

		PCR by 12 months			
		n	≤0.1%	>0.1%	
PCR by 3 months	≤10%	183 (%)	98 (53.5)	43 (23.5)	p<0.001
	>10%		6 (3.3)	36 (19.7)	
PCR by 6 months	<1%	181 (%)	93 (51.4)	36 (19.9)	p<0.001
	≥1%		9 (5.0)	43 (23.7)	
		PCR by 18 months			
		n	≤0.1%	>0.1%	
PCR by 3 months	≤10%	158 (%)	100 (63.3)	27 (17.1)	p<0.001
	>10%		11 (7.0)	20 (12.6)	
PCR by 6 months	<1%	157 (%)	97 (61.8)	22 (14)	p<0.001
	≥1%		14 (8.9%)	24 (15.3)	
		MR4.0			
		n	≤0.01%	>0.01%	
PCR by 3 months	≤10%	221 (%)	90 (40.7)	74 (33.5)	p<0.001
	>10%		8 (3.6)	49 (22.2)	
PCR by 6 months	<1%	206 (%)	86 (41.8)	48 (23.3)	p<0.001
	≥1%		12 (5.8)	60 (29.1)	
		MR4.5			
		n	≤0.0032%	>0.0032%	
PCR by 3 months	≤10%	221 (%)	70 (31.7)	94 (42.5)	p<0.001
	>10%		7 (3.2)	50 (22.6)	
PCR by 6 months	<1%	206 (%)	68 (33)	66 (32)	p<0.001
	≥1%		9 (4.4)	63 (30.6)	

* Only considered *BCR::ABL1* results of samples collected at 3, 6, 12, or 18 months with a margin of one month for less or more from these time points

Concerning the different *BCR::ABL1* transcripts, only the e14a2 transcript impacted the molecular response at 3 and 6 months (p=0.01 and p=0.03, respectively). There was no difference in the time to reach MMR, MR4.0, and MR4.5, as well as for PFS, EFS, and OS (data not shown).

### Survival analysis

**Fig. 4:**
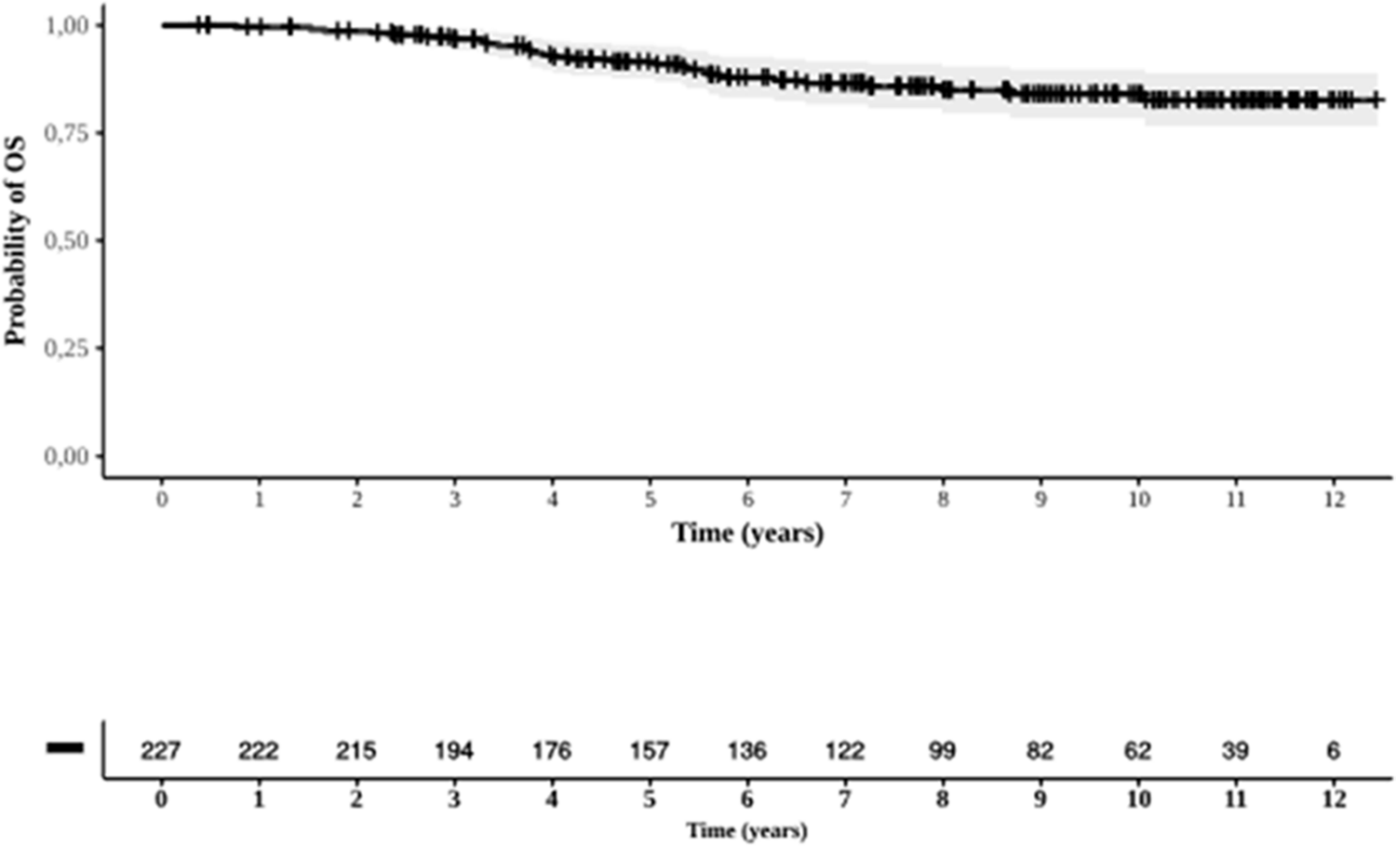
Overall survival of 227 patients with chronic myeloid leukemia

Overall, the 12.4-year OS of the 227 patients with a median follow-up time of 7.3 years was 85.8% (Figure 4).

**Fig. 5:**
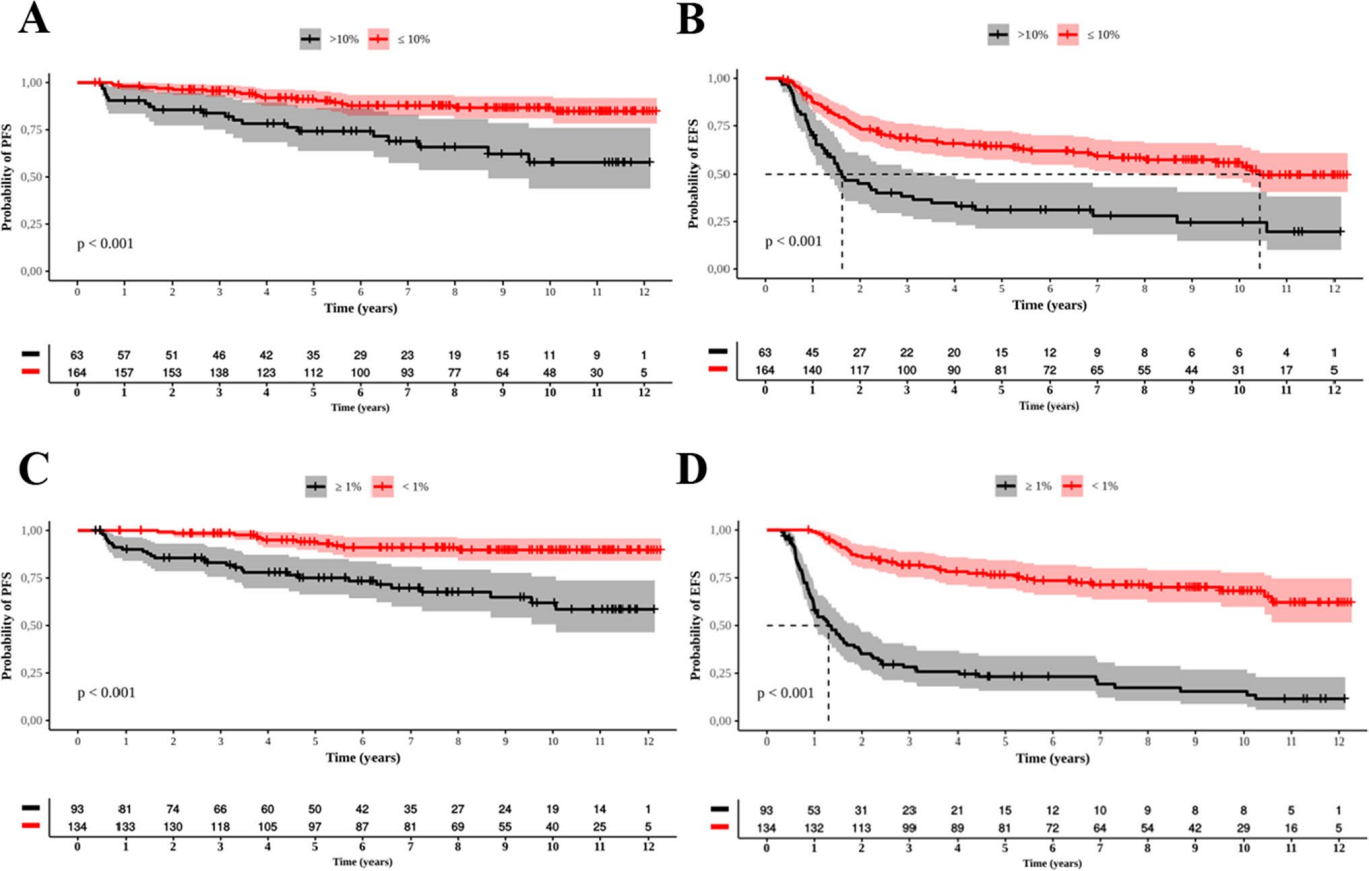
Probabilities of progression-free survival (PFS) and event-free survival (EFS) of 227 patients treated with imatinib who reached EMR by 3 and 6 months. **A**: PFS and MR by 3 months  10% and  10%; **B:** EFS and MR by 3 months  10% and  10%; **C**: PFS and MR by 6 months 1% and  1%; **D**: EFS and MR by 6 months 1% and  1%. Red and black curves represent respectively those who did and did not reach EMR

By 3 months, the mean time of PFS was 11 years (95% CI: 10.6-11.5) for patients with an EMR and 8.9 years (95% CI: 7.7-10.1) for those with *BCR::ABL1* transcript levels >10% (p<0.001, Figure 5A). The EFS was 7.9 years (95% CI: 7.1-8.7) vs. 4.4 years (95% CI: 3.2-5.6), respectively (p<0.001, Figure 5B). No difference in OS was seen between patients who achieved EMR by 3 months and those who did not - 11.2 years (95% CI: 10.7-11.7) vs. 10.6 years (95% CI: 9.7-11.5).

**Fig. 6:**
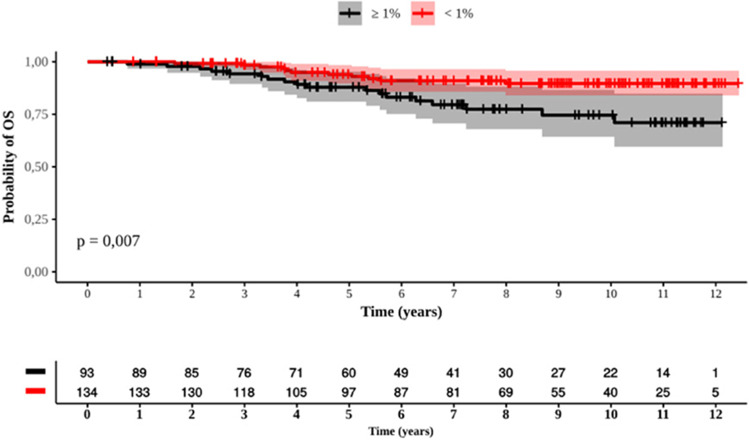
Overall survival of MR by 6 months (1% and  1%). Red and black curves represent respectively who did and did not reach EMR

Among patients who achieved molecular responses by 6 months, the PFS and EFS significantly differed between patients with <1% *BCR::ABL1* transcript levels and those with≥ 1% (Figure 5C and D). The PFS was 11.4 years (95% CI: 11-11.9) for patients with an EMR and 9 years (95% CI: 8-10) for those with *BCR::ABL1* transcript levels ≥ 1% (p<0.001). The EFS was 9.3 years (95% CI: 8.5-10.1) vs. 3.4 years (95% CI: 2.5-4.2), respectively (p<0.001). The OS for patients who had <1% *BCR::ABL1* transcript levels was superior to that of patients who had ≥ 1% transcript levels by 6 months of treatment: 11.5 years (95% CI: 11-11.9) vs. 10.3 years (95% CI: 9.5-11.1) (p=0.007) (Figure 6).

**Fig. 7:**
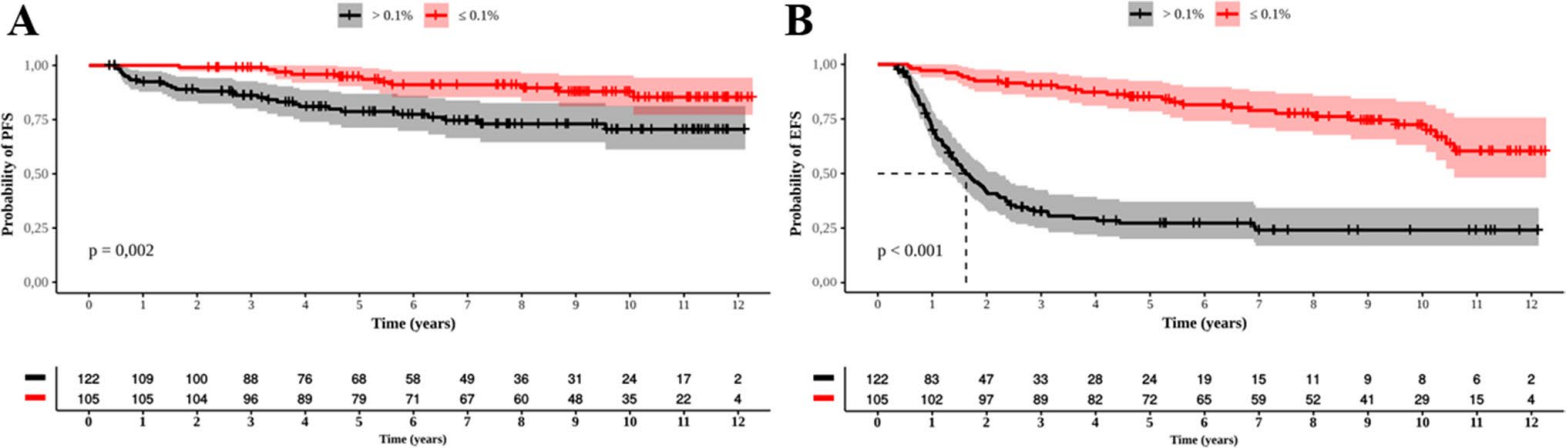
Probabilities of Progression Free Survival (PFS) and Event Free Survival (EFS) of 227 patients who reached MMR by 12 months. **A**: PFS and MMR by 12 months; **B:** EFS and MMR by 12 months. Red and black curves represent respectively who reached and did not reach MMR

Patients who achieved a MMR at 12 months presented with a PFS of 11.3 years (95% CI: 10.8-11.8), while those who did not achieve a MMR at that time presented with a PFS of 9.7 years (95% CI: 8.9-10.5) (p=0.002) (Figure 7A). The EFS was 9.9 years (95% CI: 9.1-10.6) and 4.2 years (95% CI: 3.32-5.1) (p<0.001) for patients who did and did not achieve a MMR, respectively, at 12 months (Figure 7B). No difference was seen between those who did or did not achieve a MMR at 12 months regarding OS (data not shown).

**Fig. 8:**
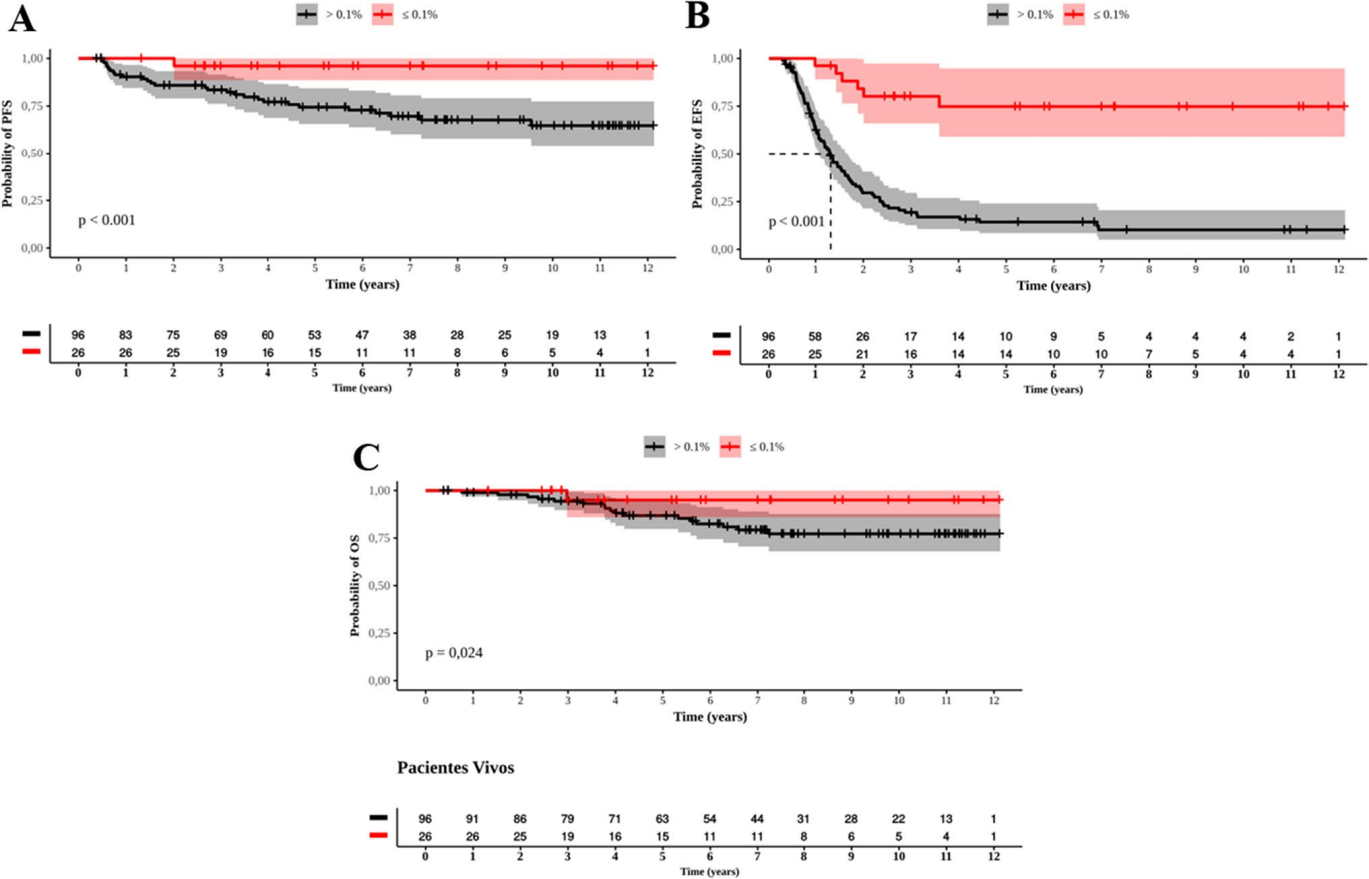
Probabilities of progression-free survival (PFS), event-free survival (EFS), and overall survival (OS) for 122 patients who only reached MMR by 18 months. **A**: PFS; **B:** EFS; **C**: OS. Red and black curves represent respectively those who reached and did not reach MMR

The PFS for those who reached a MMR at 18 months was 11.7 years (95% CI: 11-12.5), while patients who did not reach a MMR had a PFS of 9.1 years (CI 95%: 8.1-10) (p<0.001) (Figure 8A). The EFS was 9.5 years (95% CI: 7.8-11.3) and 2.7 years (95% CI: 1.9-3.4, p<0.001), respectively, for those who did and those who did not achieve a MMR at 18 months (Figure 8B). The OS was better for patients who achieved a MMR at 18 months than for those who did not, 11.6 years (95% CI: 10.8-12.5) vs. 10.3 years (95% CI: 9.6-11.1), respectively (p=0.024) (Figure 8C).

**Fig. 9:**
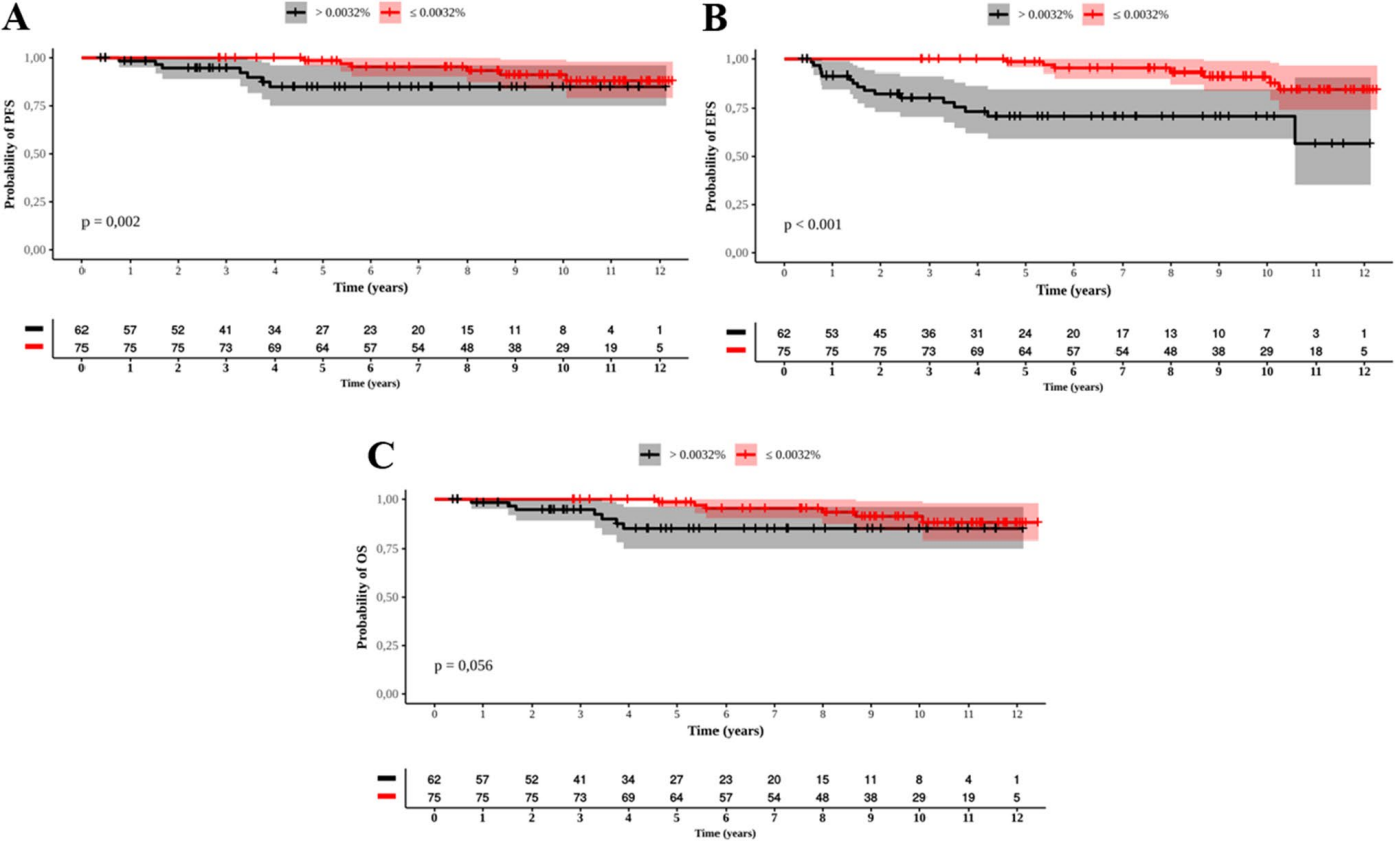
Probabilities of Progression Free Survival (**A**), Event Free Survival (**B**), and Overall Survival (**C**) in 137 patients treated only with imatinib and presented MR4.5. Red and black curves represent respectively those who reached and did not reach MR4.5

When analyzing the importance of MR4.5 in patients who were treated only with IM, we observed that the PFS of patients who reached MR4.5 was 11.6 years (95% CI: 11.2-12.1) vs. 10.8 years (95% CI: 9.9-11.8) of those who did not (p=0.002) (Figure 9A). The EFS was 11.6 years (95% CI: 11.1-12) vs. 9 years (7.7-10.3 years) (p<0.001), respectively (Figure 9B), and the OS was 11.7 years (95% CI: 11.3-12.2) vs. 10.9 years (95% CI: 9.9-11.8) (p=0.056), respectively (Figure 9C).

## Discussion

As in the Brazilian medical literature [13], the current study corroborated the median age at diagnosis of CML being 49.6 years. It may reflect the lower age of our population pyramids compared to America or Europe, as seen in Asia and Africa [3]. The patient’s age plays an important role in the treatment decision because OS, comorbidities, and the development of complications are all age-related. Patients younger than 50 are expected to live 30 more years, and therapy discontinuation is one of the principal goals [3, 4]. Most patients have the e14a2 *BCR::ABL1* transcript type, which is implicated in EMR at 3 and 6 months but without any other impact in our analyses. Publications concerning the *BCR::ABL1* transcript type are heterogenous [14–18].

To compare the collected data of this retrospective study and analyze the effectiveness and feasibility of first-line IM in daily clinical practice, we extrapolated published evidence of prospective randomized studies in which we know that the response rates in the IM group at each time point were calculated in the intention-to-treat population [19–24] (Table 4). We verified that the proportions of patients who achieved an EMR by 3 and 6 months were higher than in those studies. They had a significantly greater probability of attaining a MMR by 12 and 18 months and later reaching MR4.0 and MR4.5 than those who did not get the same benchmarks. EMR by 3 months and MMR by 12 months did not show advantages in PFS and OS but did show a significant difference in EFS. Patients with *BCR::ABL1* transcript levels < 1% at 6 months and MMR at 18 months showed an impact in the PFS and OS, as seen in the CML-IV study [19]. Like the IRIS study [22], reaching MMR at 12 months does not interfere with OS when all deaths are included, regardless of the CML-related deaths, differently from getting MMR at 18 months. An attempt to treatment discontinuation can be considered if sustained DMR of sufficiently long duration has been achieved. The younger the patient, the stronger the case for achieving TFR [3]. Patients who achieved a deep MR had a statistically significant advantage in PFS and EFS and a trend toward better OS. Most patients achieve MMR in the first two years of treatment and a deep MR in the second to the fourth year. For young patients without morbidities or women wanting to become pregnant, the goal is to reach deep MR (MR4.0 and MR4.5). Thus, achieving an EMR in 3 and 6 months is significantly important.

**Table 4:** Molecular response, progression-free survival, and overall survival in patients treated with first-line imatinib mesylate

Clinical Trial	*BCR::ABL1* ≤10% by 3 months	*BCR::ABL1* <1% by 6 months	*BCR::ABL1* ≤0,1% by 12 months	Total MMR (%)	Total MR4.5 (%)	PFS (%)	OS (%)	IM by end of study (%)	Follow-up (years)
This study	74.2	65	56.5	89.8	51.1	91	91	60.8	7.3
IRIS^22,23^	-	-	53	93.1	63.2	80	83,3	48.3	10.9
IV CML^19^	68.5	61	36.7	92.2	67.2	80	80	62	9.5
TIDEL^24^			47	73		93	94		2
DASISION^20^	64		28	64	33	86	90	63	5
ENESTnd^21^	67		27	60.4	31.4	91	91.7	49.8	6
Bendit et al.^11^			37.9	80.5	25	95	94.2		5

**Fig. 10:**
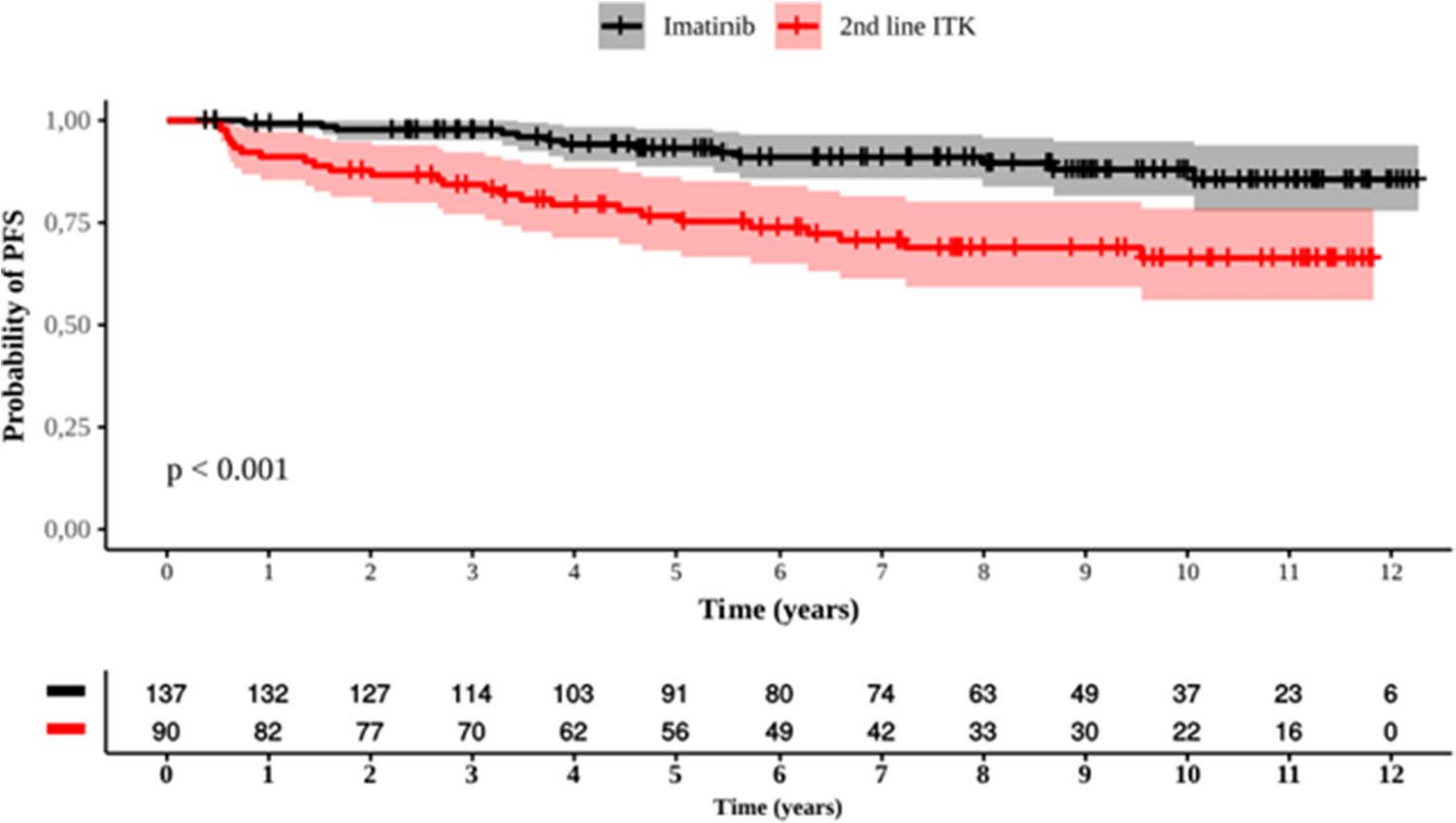
Progression-free survival (PFS) of patients treated only with IM (black curve), and those who switch to second-line treatment (red curve)

The second-line therapy could be started at any time, but in practice, most switches occur between the third month and the first year of treatment, and the most frequent causes are resistance and intolerance to IM [25]. Although switching to a second-line inhibitor occurred in 39.7% of patients, 60.3% (137/227) were still using IM at the end of the study. 86.7% of patients chose dasatinib as the second-generation inhibitor, previously approved than nilotinib in Brazil. The median time to change the TKI was 1.2 years. Achieving an EMR at 3 and 6 months mitigates the need to switch to second-line treatment. Another important point was that patients who continued to be treated with IM had a higher PFS than patients who changed TKIs (p<0.001), while OS was not affected (Figure 10).

As the life expectancy of patients with CML is close to that of the general population, OS is not one of the best outcomes for comparing the results. Failure to achieve MMR has been widely accepted as a warning sign of treatment failure and grounds for therapeutic changes. However, a lack of data shows that acting upon this outcome improves clinically relevant endpoints like OS. The current practice is switching CML patients to more expensive and toxic therapy when MMR milestones are unmet [26].

In conclusion, the EMR correlates with higher rates of deeper molecular responses and possible TFR. Since adherence to life-long TKI therapy is critical for most patients with CML, TKI costs and cost-effectiveness have become crucial issues for patients and society, which are justifiably involved in drug costs [3]. OS correlated only with *BCR::ABL1* transcript levels < 1% at 6 months (equivalent to CCyR) and MMR at 18 months, demonstrating that in real life, CCyR is still the primary marker of survival and the MMR does not necessarily need to be early. The results presented by this Brazilian cohort are comparable to those described in prospective and randomized studies, and IM proved to be an excellent therapeutic choice with a known and tolerable side-effect profile and a lower financial cost, especially for our public health system, which covers 75% of the Brazilian population.

The present study has some limitations, such as its retrospective nature and the lack of risk classification, such as that performed by Sokal [27], EUTOS [28], and the ELTS studies [29] that could somehow help in understanding the reason for molecular responses in the predetermined milestones that were not reached according to the European Leukemia Net [3, 12] or the NCCN [30]. The presented results must be extrapolated with caution since this is a unicentric study where the regular molecular monitoring of *BCR::ABL1* transcript levels is a non-reproducible reality in most centers.
