# Correction to: Real‑world imatinib mesylate treatment in patients with chronic myeloid leukemia: The importance of molecular monitoring and the early molecular response

**DOI:** 10.1007/s00277-023-05242-1

**Published:** 2023-05-09

**Authors:** Amanda Pifano Soares Ferreira, Fernanda Salles Seguro, Andre Ramires Neder Abdo, Fernanda Maria Santos, Felipe Vieira Rodrigues Maciel, Luciana Nardinelli, Ricardo Rodrigues Giorgi, Antonio Roberto Lancha Ruiz, Milton Pifano Soares Ferreira, Eduardo Magalhaes Rego, Vanderson Rocha, Israel Bendit

**Affiliations:** 1 Hematologica Clinic Oncoclinicas, Belo Horizonte, Brazil; 2 Department of Hematology, Transfusion and Cell Therapy, University of Sao Paulo Medical School (HCFMUSP), Sao Paulo, Brazil; 3 Department of Hematology, Cancer Institute of Sao Paulo, University of Sao Paulo Medical School (ICESP), Sao Paulo, Brazil; 4 Laboratory of Medical Investigation in Pathogenesis and targeted therapy in Onco‑Immuno‑Hematology (LIM/31), Department of Hematology, Hospital das Clínicas HCFMUSP, Faculdade de Medicina, Universidade de Sao Paulo, Sao Paulo, Brazil; 5 Minas Gerais Federal University, Belo Horizonte, Brazil; 6 Hemato‑Oncologia, DASA-Genômica, Sao Paulo, Brazil

**Correction to: Annals of Hematology**

10.1007/s00277-023-05189-3

In the published paper, almost all the figures and tables of the article (Table 1, table 2, figure 3, figure 4, figure 5 and figure 6 are incompatible with its current captions.

The original article has been corrected.

